# Knockout of the Transducin-Like Enhancer of Split 6 Gene Affects the Proliferation and Cell Cycle Process of Mouse Spermatogonia

**DOI:** 10.3390/ijms21165827

**Published:** 2020-08-13

**Authors:** Meiying Feng, Yinshan Bai, Yun Chen, Kai Wang

**Affiliations:** 1 College of Life Science, Zhaoqing University, Zhaoqing 526061, China; jony.ya@163.com; 2 National Engineering Research Center for Breeding Swine Industry, Guangdong Provincial Key Lab of Agro-Animal Genomics and Molecular Breeding, College of Animal Science, South China Agricultural University, Guangzhou 510642, China; xuefei200403@163.com; 3 Henry Fok College of Biology and Agriculture, Shaoguan University, Shaoguan 512000, China; luxixiworld@163.com

**Keywords:** mouse, *Tle6*, gene knockout, cell proliferation, cell cycle

## Abstract

*Tle6* (Transducin-like enhancer of split 6) is a member of the Tle co-repressor superfamily, which is expressed in various tissues of invertebrates and vertebrates and participates in the developmental process. However, the current research has only found that the TLE6 mutation is related to infertility, and the key regulatory mechanism of TLE6 remains to be explored. In this study, we combined Clustered Regularly Interspaced Short Palindromic Repeats (CRISPR)-Cas9 and the Tet-on system to construct mouse spermatogonia cell lines that induced TLE6 protein knockout (KO), and studied the effect of *Tle6* on mouse spermatogonia proliferation and the cell cycle. The results showed that, after drug induction, the *Tle6* gene in mouse spermatogonia was successfully knocked out at the genome and protein levels, and the *Tle6* gene knockout efficiency was confirmed to be 87.5% with gene-cloning technology. At the same time, we also found that the mouse spermatogonia proliferated slowly after the *Tle6* knockout. Using flow cytometry, we found that the cells did not undergo significant apoptosis, and the number of cells in the S phase decreased. After real-time quantity PCR (qRT-PCR) analysis, we found that the expression of cell-proliferation-related genes, CCAAT enhancer-binding protein α(*C/ebp α*), granulocyte-colony stimulating factor(*G-csf)*, cyclin-dependent kinases 4(*Cdk 4*), Cyclin E, proliferating cell nuclear antigen(*Pcna*), and S-phase kinase-associated protein 2 (*Skp* *2*) was significantly reduced, which further affected cell growth. In summary, Tle6 can regulate spermatogonia cell proliferation and the cell cycle and provide a scientific basis for studying the role of TLE6 on spermatogenesis.

## 1. Introduction

Transducin-like enhancer of split (TLE) proteins are the mammalian homologs of Groucho, which is a conserved family of co-repressors that exist in animals. In nature, such proteins are expressed in invertebrates (flies) and vertebrates (mice and humans). In mammals, the Groucho family has six main member genes (*Tle1–6*). Among these, *Tle1–4* are full-length genes, *Tle5* and *Tle6* are two short-type genes that can inhibit the function of the *Tle1–4* genes, and there is a close relationship between the short-type genes and full-length genes. The TLE5 protein only includes two regions located at the amino terminus, which play key roles in the processes of bone growth and cell apoptosis. After knocking out TLE5 in mice, researchers found that the bones of the mice grew slowly, and short-term growth retardation occurred [1,2].

In a study of human cerebellar granule neurons, TLE5 inhibited the TLE1-mediated anti-apoptotic effect and promoted cell apoptosis [3]. The transducin-like enhancer of split 6 (*Tle6*), a member of the Tle transcriptional co-repressor family, was first identified as the target of hepatic leukemia factor (E2a) and is widely expressed in mouse embryos and adult tissues [4]. Apart from its shorter length, its sequence is very different from other Groucho/Tle family members. The mouse TLE6 protein contains 581 amino acids with a molecular weight of 65 kDa. Unlike typical TLE1–4 proteins, TLE6 has only five tryptophan-aspartic acid (WD) repeat units [5], missing two that are involved in the Groucho/TLE oligomerization, in binding to specific transcription factors (amino-terminal TLE N-terminal (also known as the Q-enrichment domain)), and that mediate and participate in the interaction of most transcription factors (carboxy-terminal WD40 repeat domain) [6]. As most of the amino-terminal domains are missing, *Tle6* may not be able to form heterodimers with other *Tle* genes and may interact with other proteins alone.

The *Tle6* gene is highly expressed in the ovaries of newborns, and the TLE6 protein combines with a variety of maternal effector proteins to form the maternal effector complex necessary for the early embryonic development of mammals (the subcortical maternal complex, SCMC), which regulates early embryonic development and cell division in mice and sheep [7,8]. Early research found that *Tle6* is a maternal effector gene required for the cleavage stage of embryogenesis and that the TLE6 protein is a protein necessary for the formation of SCMC in mouse oocytes and early embryos. The TLE6 protein controls the symmetrical division of fertilized mouse eggs by regulating the actin cytoskeleton. Knocking out TLE6 causes infertility in mice and [9] humans [10], which greatly hinders the study of *Tle6* gene function.

The above research implies that TLE6 plays a key role in reproductive development, and performs key regulatory functions at various time-points in cell and embryo development. To date, the majority of studies have been based on exogenous TLE (overexpression/misexpression) based on cell culture and animal analysis; however, the study of endogenously expressed TLE is more reliable and important. As the expression of TLE during development is limited by time nodes, the study of endogenously expressed TLE is particularly important. Therefore, the present study combined the CRISPR-Cas9 system and the Tet-on system to conditionally induce the knockout of the mouse spermatogonia TLE6 protein to construct a mouse spermatogonia cell line that can induce *Tle6* gene knockout, to provide a cell model for further study on the function of the *Tle6* gene in spermatogenesis.

The mammalian cell cycle is an important process in cell life and includes four different stages (G0/G1, S, G2, and M). The progression of each stage is affected by cyclin-dependent kinase (CDK) and cyclin (cyclin) co-regulation [11]. In the advancement of the cell cycle, cyclin and CDK form a cyclin–CDK complex that affects cell proliferation. Researchers have indicated that the cyclin D–CDK 4 complex controls cells in the G0/G1 phase, while the cyclin E–CDK 2 complex controls cells from the G0/G1 phase to the S phase [12]. In the current research, the role of *Tle6* on spermatogonia cell proliferation and the cell cycle was investigated using an immortalized mouse spermatogonia cell line, C18-4. First, we constructed KO cell lines via the CRISPR-Cas9 gene-editing method to explore the role of *Tle6* in C18-4. A series of cellular and molecular experiments showed that *Tle6* deficiency inhibited cell proliferation through G1–S cell cycle arrest and decrease cell motility. The regulation of cell-cycle proteins might cooperate in the altering of cell physiological processes. Our study revealed a function of *Tle6* involved in C18-4, and provides a scientific basis for studying the role of TLE6 in spermatogenesis.

## 2. Results

### 2.1. CRISPR/Cas9-Mediated KO of Tle6 in Spermatogonia

**Figure 1:**
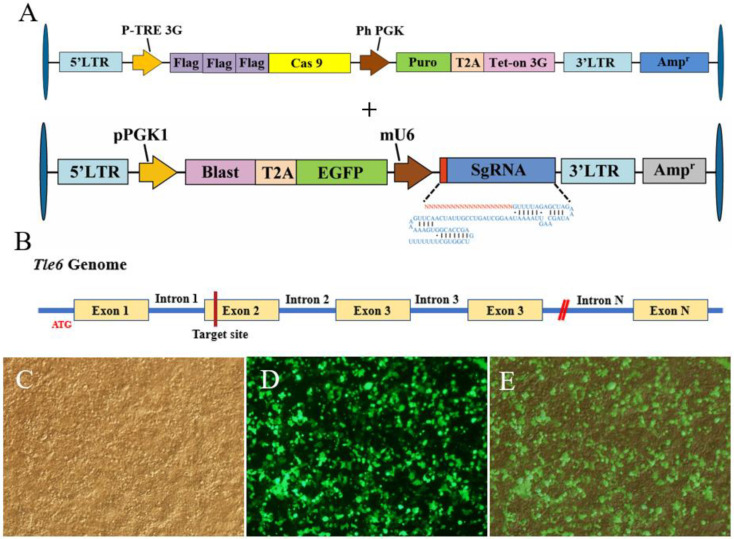
plvx–Tet3G–Cas9 and plvx–Tle6–SgRNA–EGFP lentiviral packaging. (**A**) plvx–Tet3G–Cas9 (top) and plvx–mU6-Tle6–SgRNA–EGFP (bottom) vector linear map, oval means vector junction, arrow means promoter, “+” means “and”; (**B**) Tle6–SgRNA gene target location (ATG is the start codon, the blue line is the intron sequences. The orange frame is the exon sequence, the red vertical line is the position of the mouse genome where the target sequence is located, and the double diagonal, vertical line is the omitted part of the exon and intron downstream in the mouse genome); (**C**) plvx–mU6-Tle6–SgRNA–EGFP lentivirus-packaged 293 FT white-light image; (**D**) lentivirus-packaged 293 FT fluorescence image; (**E**) merged images of C and D.

To obtain a mouse spermatogonia knockout of the *Tle6* gene, we infected the cells with a Tet3G–Cas9 virus solution (Figure 1A). After the mouse spermatogonia stably expressed CAS9, the SgRNA target sequence was selected in the exon of *Tle6* (the results are not shown). We found that an active sequence was obtained in the second exon (Figure 1B). We used PCR to amplify the 375 bp mU6 and 163 bp Tle6–SgRNA fragments, and used fusion PCR technology to obtain the target fragment mU6–Tle6–SgRNA (the results are not shown). Subsequently, the empty vector and target fragment were digested by Nhe I and Xho I, and a plvx–Tle6–SgRNA–EGFP shuttle vector was successfully constructed using gene-cloning technology (Figure 1A). When packaging the Tle6–SgRNA virus solution, because the vector has a fluorescent label (Figure 1D), the lentiviral packaging plasmids pMD2.G and psPAX2 and the shuttle vector were co-transfected with 293FT cells at a certain ratio, and the green fluorescent protein was observed at 48 h. The expression (Figure 1C,E), transfection efficiency reached more than 80%.

**Figure 2:**
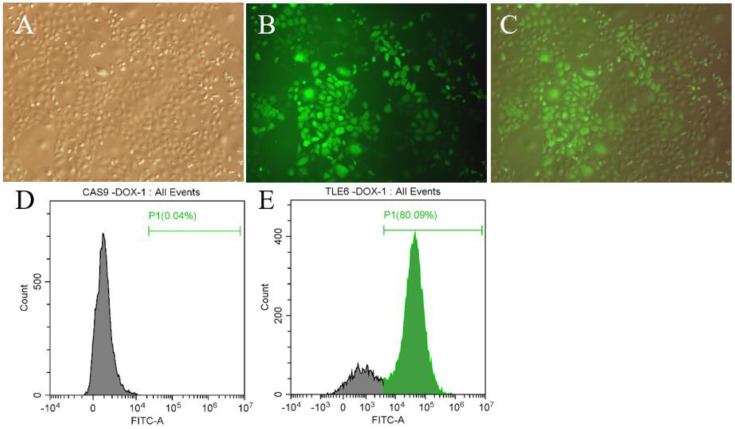
The detection of fluorescent protein expression in mouse spermatogonia. (**A**,**B**): White light and fluorescence of mouse spermatogonia stably expressing mU6–Tle6–SgRNA; (**C**) merged results of A and B; (**D**) Flow-cytometry detection of the fluorescent protein expression of mouse spermatogonia before infection (gray is the number of negative cells and green is the number of positive cells); (**E**): flow-cytometry detection of the fluorescent protein expression in infected mouse spermatogonia (gray is the number of negative cells and green is the number of positive cells).

In order to construct *Tle6*-knockout mouse spermatogonia, we infected the mouse spermatogonia stably expressing CAS9 with Tle6–SgRNA virus liquid, and, after subjecting cells to Blast drug screening, the expression of green fluorescent protein in the cells was detected with a fluorescence microscope (Figure 2A–C). Subsequently, we used flow cytometry to further detect the expression of fluorescent protein expression of cells, and found that the cell infection rate was high, reaching 80% (Figure 2D,E). This shows that we successfully obtained mouse spermatogonia stably expressing plvx–mU6–Tle6–SgRNA–EGFP.

### 2.2. Detection of the Effect of Tle6 Knockout in Mouse Spermatogonia

**Figure 3:**
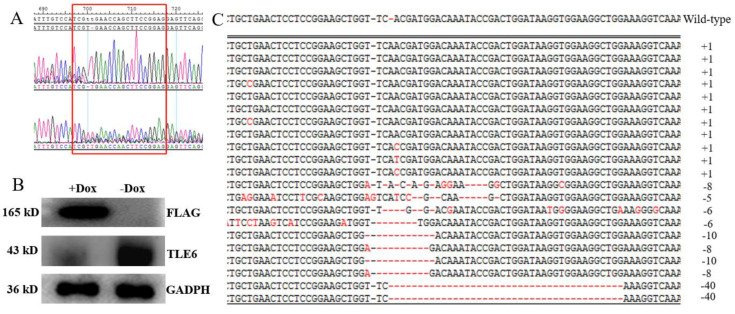
Detection of knockout of *Tle6* in mouse spermatogonia. (**A**): The *Tle6*-KO genome sequencing analysis results after Dox induction (the red box is the base of Tle6–SgRNA target sequence); (**B**): Western blot detection of TLE6 protein expression after Dox induction *Tle6*-KO genome sequencing analysis results (–Dox represents the spermatogonia of mice without gene knockout and without the inducer doxycycline control group, and +Dox represents the spermatogonia of mice with the *Tle6* gene knockout added to the doxycycline test group); (**C**): Analysis of the knockout efficiency of the spermatogonia *Tle6* gene (the wild-type sequence at the top is the standard sequence, and the sequence between the two lines is the sequencing sequence. The black horizontal lines indicate points where there were no bases in the standard genome. The red horizontal lines indicate bases in the standard genome, if there were none, the bases are knocked out, and the red bases are the mutant bases. The numbers on the right are the results of the base alignment between the sequence and the standard sequence, i.e., the number of bases knocked out relative to the standard sequence, where the negative number is the number of bases knocked out relative to the standard sequence.

To further verify the *Tle6* gene knockout results, we amplified the genomic fragments near the mouse spermatogonia *Tle6* target sequence (CTCCGGAAGCTGGTTCACGA) for sequencing and analyzed the peak map of the target. The sequencing results showed that the *Tle6* gene in mouse spermatogonia was successfully knocked out, and a peak appeared near the target sequence (Figure 3A).

Subsequently, to detect the expression of TLE6 in mouse spermatogonia, Western blotting identified inhibition of the expression of TLE6 at the protein level (Figure 3B). To further analyze the knockout efficiency, after the *Tle6* knockout, the above target fragment was ligated with the pMD18-T vector and sequenced. We found that 11 genes were inserted, 10 genes were deleted (Figure 3C), and three genes were present for the wild type. The efficiency of the *Tle6* gene knockout reached 87.5% (Figure 3C). The results of these data confirmed that we successfully obtained *Tle6*-KO mouse spermatogonia.

### 2.3. The Effects of the Tle6 Gene Knockout on the Proliferation of Mouse Spermatogonia

**Figure 4:**
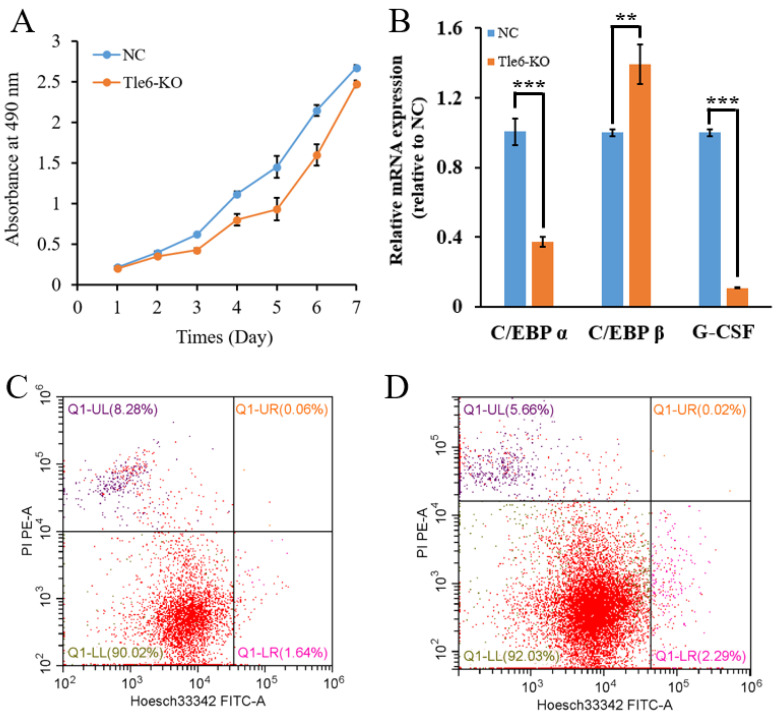
The effects of *Tle6* KO on the proliferation of mouse spermatogonia. (**A**): Cell growth curve before and after the knockout of mouse spermatogonia *Tle6* (blue NC is the non-specific control, orange *Tle6*-KO is the experimental group (the same below), the x-axis shows the number of days of cell culture, and the y-axis is at 490 mm absorbance; ** means *p* < 0.01 and *** means *p* < 0.001); (**B**): the expression of cell-proliferation-related genes before and after the knockout of mouse spermatogonia Tle6 (x-axis is the relative expression of the mRNA value, and the y-axis label is the name of the gene related to mouse cell proliferation); (**C**): the detection of apoptosis before the knockout of mouse spermatogonia *Tle6* (x-axis is Hoesch33342 staining, the y-axis is 3,8-Diamino-5-[3-(diethylmethylammonio)propyl]-6-phenylphenanthridinium diiodide (PI) staining (same below)); (**D**): the detection of apoptosis in mouse spermatogonia Tle6 before and after knockout.

In order to detect the effect of the *Tle6* knockout on cell proliferation, we used the 3-(4,5-dimethylthiazol-2-yl)-5-(3-carboxymethoxyphenyl)-2-(4-sulfophenyl)-2H-tetrazolium inner salt (MTS) reagent to detect the growth of mouse cells before and after the *Tle6* knockout. After plotting the growth curve, we investigated the mouse spermatogonia after *Tle6* knockout relative to the control cells. The proliferation rate was slow (Figure 4A), which shows that the TLE6 protein affected cell proliferation.

To further study the effect of the *Tle6* knockout on mouse spermatogonia, we examined the expression of cell-proliferation-related genes and found that the expression levels of *C/EBP α* and *G-CSF* were significantly reduced, and the expression levels of *C/EBP β* were significantly increased (Figure 4B). However, the detection of apoptosis by flow cytometry found that knocking out Tle6 had no significant effect on the apoptosis of mouse spermatogonia (Figure 4C,D).

### 2.4. The Effects of Tle6 Gene Knockout on the Mouse Spermatogonia Cell Cycle

**Figure 5:**
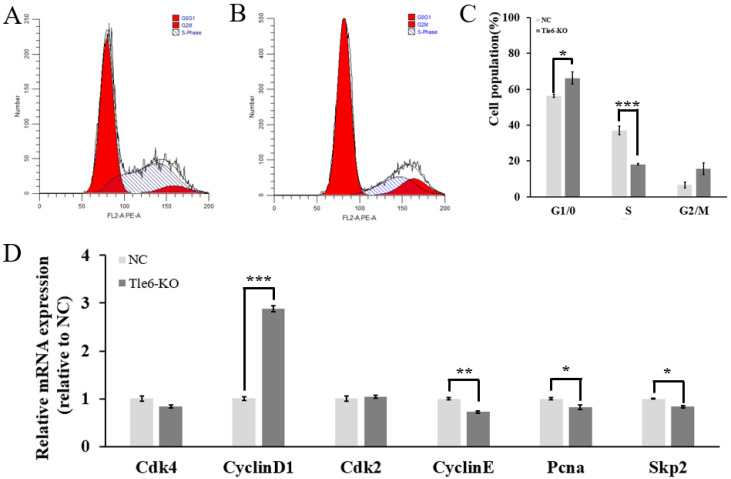
The effects of the *Tle6* knockout on the cell cycle of mouse spermatogonia. (**A**): Cell-cycle detection before the mouse spermatogonia *Tle6* knockout; (**B**): cell-cycle detection after the mouse spermatogonia *Tle6* knockout; (**C**): cell-cycle analysis before and after the mouse spermatogonia Tle6 knockout (the x-axis is the stage of the cell cycle, the y-axis is the percentage of the number of cells); (**D**): the expression of cell-cycle-related genes before and after the knockout of *Tle6* in mouse spermatogonia (the x-axis is the relative expression value of mRNA, and the y-axis is the mouse cell-cycle gene name). Note: NC is the control group, *Tle6*-KO is the experimental group; * means *p* < 0.05, ** means *p* < 0.01, and *** means *p* < 0.001.

In order to detect the effect of the *Tle6* knockout on the cell cycle, we used flow cytometry to further examine the cell cycle and found that there was a significant change in the cell cycles of mouse spermatogonia before and after the *Tle6* knockout (Figure 5A,B). Not only did the ratio of G1/G0-phase cells significantly increase (*p* < 0.05) but the ratio of the S-phase cells also significantly decreased (*p* < 0.001, Figure 5C). Studies have demonstrated that the cell-cycle-related proteins cyclin D1, cyclin E, CDK 2, and CDK 4 can form the corresponding cyclin D1–CDK 4 and cyclin E–CDK 2 complex, which affects the process of cells from the G1 phase to the S phase. PCNA and SKP2 also affect DNA synthesis in the S phase of cells. After examining the expression of mouse cell-cycle-related genes, we found that the expression of *Cdk 4*, *Cyclin E*, *Pcna*, and *Skp 2* in mouse spermatogonia was significantly reduced after the *Tle6* knockout compared to the control cells (*p* < 0.05). For cyclin, the expression level of D1 increased significantly (*p* < 0.001, Figure 5D). This implies that the *Tle6* knockout affected the progress of the mouse spermatogonia cell cycle, resulting in slow cell growth rate.

## 3. Discussion

CRISPR/Cas gene-editing technology has been a revolutionary advancement in biomedical research in recent years, which greatly enhances the process of researching functional genomics and has been widely used in the editing of various cell genomes [13]. The plvx–mU6–Tle6–SgRNA–EGFP vector used in this article carries a fluorescent label, which was used to intuitively and conveniently detect the expression of the target sequence in the cell. The Tet-on tetracycline induction system is an expression system based on the addition of Dox to induce the expression of a target gene. The Tet-on induction system has been successfully used to achieve conditional induction expression in a variety of cell populations/lines [14,15,16]. In this paper, we used the Tet-on system and the CRISPR/Cas9 system to edit the *Tle6* gene, which realized the controllability of cleavage and the conditional induction of a *Tle6* gene knockout in mouse spermatogonia. Knockout occurred, and the knockout efficiency reached 87.5%. When using the Tet-on–CRISPR/Cas9 system to edit the target gene, large-scale apoptosis of the knockout cells was avoided, and subsequent research could be carried out; in addition, the gene expression and function of the two cells before and after induced knockout was detected.

The TLE protein does not directly bind to DNA; it interacts with several DNA-binding proteins, thereby inhibiting the expression of downstream genes and proteins, and regulating life activities, neuronal differentiation, and tumorigenesis of invertebrates and vertebrates [6,17,18]. In cortical neural progenitor cells, the *Tle6* gene can interfere with cells’ differentiation into neurons [6]. In colon cancer cells, the *Tle6* gene interacts with the gastrointestinal tumor suppressor RUNX3, increasing tumor cell proliferation, colony formation, cell migration, and xenograft tumorigenesis [17]. The literature revealed, to varying degrees, that the *Tle6* gene may affect the ability of cell proliferation, differentiation, and cycle. With the discovery of, and extensive research into the core inhibitors of TLE in humans and animals, there is a consensus in the literature that TLE-mediated inhibitory effects are regulated by spatial and temporal distribution.

Sperm formation is a long and orderly process, regulated at many key time nodes. Spermatogenesis involves the mitotic cells increasing their production of spermatogonia and producing stem cells and primary spermatocytes. After knocking out *Tle6* in mouse spermatogonia, the cell proliferation rate was extremely low (Figure 4A), and the expression of cell-proliferation-related genes was significantly affected (Figure 4B). Among these, granulocyte colony-stimulating factor (G-CSF) is a member of the hematopoietic growth factor family [19] with several physiological roles, including the stimulation of hematopoietic cell proliferation, differentiation, and maturation [20].

Studies have reported that G-CSF could stimulate the proliferation of spermatogonia and protect spermatogenesis [21]. The C/EBP is an important negative regulatory protein in cell proliferation, and C/EBP-α and -β can regulate cell proliferation and differentiation by inhibiting the action of the E2F complex [22,23]. In this study, the expression levels of G-CSF and C/EBP α in spermatogonia lacking TLE6 were significantly reduced. Although the expression of C/EBP α was significantly reduced, studies indicated that the increased expression of C/EBP β could make up for the impact of C/EBP-α deficiency [24,25]. The result eventually leads to slow growth and cycle disorders in spermatogonia lacking TLE6.

*Tle6* is also expressed in various tissues of animals [26], and the expression of *Tle6* in ovarian tissue greatly affects the development of early embryos in mammals [7,8,27,28]. *Tle6* is essential for the growth and development of cells. During the transition from early embryos to two cells in mice, most of the fertilized eggs of Tle6-mutant female mice cannot cleave, causing infertility [9]. Studies have indicated that female patients with recurrent miscarriages may have mutations in the *Tle6* gene, and the phenotype is similar to *Tle6*-knockout mice. Although *Tle6* does not affect the discharge of mature oocytes from patients, it affects the termination of embryo cleavage in the cleavage stage [10]. When *Tle6* was knocked out in mouse spermatogonia, although the cell growth did not appear to stagnate, the growth rate was extremely low.

Cell proliferation involves four different stages of the cell cycle (G0/G1, S, G2, and M). The cell-cycle process is regulated by two proteins, namely cyclin and its kinase partner, cyclin-dependent kinase (cdks). The transmission of restriction points is coordinated by two cyclin families, namely the cyclin D family and the cyclin E family. The activation sequence is that the D-type cyclin binds and activates *cdk 4* and *6*, and then cyclin E activates *cdk2* [11,12]. Cyclin E targets the components of the DNA synthesis machinery, leading to the assembly of the complexes required for DNA replication [29]. S-phase kinase-related protein 2 (SKP2) cooperates with cyclin D and E to regulate cell-cycle progression in the G1–S phase [30].

The overexpression of cyclin D and E and SKP2 occurs in most types of malignant cancers and is significantly related to excessive cell proliferation. Members of this family are prognostic markers of pancreas, endometrial, and head and neck tumors [31,32]. Proliferating cell nuclear antigen (PCNA) is a multifunctional protein present in the nuclei of eukaryotic cells that plays an important role as a component of the DNA replication machinery, as well as in DNA repair systems. In recent decades, researchers have proven that a large number of proteins related to DNA replication, DNA repair, cell-cycle control, epigenetic regulation, cell survival, and cell metabolism interact with PCNA. Blocking PCNA could inhibit DNA replication [33]. In this study, the expression of cyclin D1 in spermatogonia lacking TLE6 was significantly increased, the expression of *CDK4* was significantly decreased, and cyclin E, which ultimately regulates DNA replication and synthesis, was significantly decreased, which ultimately prevented the cell from entering the S phase from G1. During this period, the expression of *Skp2* and *Pcna* was significantly downregulated. This may have been due to the lack of TLE6 in the process of *cyclin D* regulating *cyclin E*, resulting in the inability to activate *cyclin E* normally and block DNA replication. This requires further experiments to verify.

## 4. Materials and Methods

### 4.1. Cell Culture and CRISPR/Cas9-Mediated KO of Tle6 in Spermatogonia

The lentiviral packaging plasmids pMD2.G and psPAX2 were purchased from Invitrogen (Carlsbad, CA, USA), while the lentiviral doxycycline-inducible (Dox) FLAG-Cas9 (50661) and U6-sgRNA vector (50662) plasmids were purchased from Addgene (Watertown, MA, USA). The targeting sgRNA of *Tle6* was designed, as previously described [34], and constructed for the plvx–mU6-Tle6–SgRNA–EGFP vector using gene-cloning technology (see Supplementary Table S1 for the related primer sequences); All constructs were confirmed by sequence analysis. Next, 24 μg of DNA, with the proportions of pMD2.G, psPAX2, and shuttle plasmid (plvx–Tle6–SgRNA–EGFP or plvx–Tet3G–Cas9) set at 2:1:2, was cotransfected into 293 FT cells, with 70–80% confluence, using the Lipofectamine3000 transfection reagent (Life Technologies, Waltham, CA, USA), and cultured in a cell incubator at 37 °C, 5% CO_2_ to produce lentivirus of FLAG–Cas9. The virus-containing supernatant was collected at 48 h and 72 h after transfection, and the FLAG–Cas9 virus solutions were concentrated using Millipure ultrafiltration tubes, Amicon^®^ Ultra-15 10K centrifugal filter devices (Merck Millipore, Darmstadt, Germany), at 4000× *g* for 30 min at 4 °C. The same protocol was used to produce the Tle6–SgRNA virus.

The mouse spermatogonia were obtained from Prof. Wenxian Zeng (School of Animal Science and Technology of Northwest A&F University, Shanxi, China) and cultured in Dulbecco’s modified Eagle’s medium (DMEM) (Gibco, Carlsbad, CA, USA) supplemented with 10% (vol/vol) fetal bovine serum (FBS) at 37 °C and 5% CO_2_. Subsequently, the FLAG–Cas9 concentrated lentivirus was co-infected with 10 μg/mL Polybrene. After 12 h, the lentivirus was removed and mouse spermatogonia cells were selected with 2 µg/mL puromycin for five days to seven days to screen mouse spermatogonia that stably expressed Cas9. These cells were then used to infect concentrated Tle6–SgRNA lentivirus via the same protocol, except for the selection with 10 μg/mL BLAST. The expression of fluorescent cells before and after the infection was then observed and detected with a fluorescence microscope (TH4-200, Olympus, Tokyo, Japan) and flow cytometry (CytoFLEX, Beckman Coulter, Brea, CA, USA), and we calculated the proportion of fluorescent cells. This trial has been approved by the Bioethics Committee of Zhaoqing University (FC-1013).

### 4.2. Target Gene Amplification

The mouse spermatogonia genomes were investigated before and after *Tle6*-KO was extracted, and PCR was conducted for the sequence near the Tle6–SgRNA target in a programmed thermal cycler (Bio-Rad, Hercules, CA, USA) (primer sequences are shown in Supplementary Table S1). The following PCR conditions were used: initial denaturation at 94 °C for 5 min, followed by 35 cycles of denaturation at 94 °C for 30 s, annealing at 60 °C for 30 s, and extension at 72 °C for 1 min, and then a 7 min final extension at 72 °C. Subsequently, a part of the amplified fragment was compared with the sequence published on NCBI to analyze the effect of the *Tle6* knockout using EditSeq software (DNAStar, Madison, WI, USA). Another part of the fragment was ligated into the PMD18-T vector and amplified. We determined the sequence (primer sequences are shown in Supplementary Table S1), and then the SeqMan software (DNAStar) was used to detect the *Tle6* knockout efficiency of the amplified sequence.

### 4.3. Western Blot Analysis

A whole-cell lysis assay (KGP250, KeyGEN BioTECH) was used to extract the total protein of the mouse spermatogonia before and after Tle6-KO, and the BCA Protein Quantitation Assay (KGP250, KeyGEN BioTECH, Nanjing, Jiangsu, China) was used to detect the protein concentration with a microplate reader, Synergy2 (BioTek, Winooski, VT, USA). The extracted proteins (15 μg) were loaded on a 10% separation gel and 4% concentration gel (electrophoresis conditions: concentrated gel constant pressure 70 V, 40 min; separation gel constant pressure 90 V, 2 h) and then transferred onto polyvinylidene difluoride (PVDF) membranes (Hybond-P, Helsinki, Finland) using a low-temperature wet transfer (the membrane transfer condition was a constant pressure of 80 V for 1 h), and nonspecific binding was blocked for 1 h using DPBS (Gibco) buffer with 5% nonfat dry milk at room temperature. According to the size of the protein marker and the target protein, TLE6 (1:500), GADPH (1:1000), and FLAG (1:1000) antibodies (see Supplementary Table S2 for antibody details) were added to incubate the blots at 4 °C overnight. The washing protocol of the hybridization membrane was then performed, as previously reported [35]. After that, the blots were added to the corresponding diluted anti-source IgG (1:2000) and incubated at room temperature in the dark for 1 h. The blots were then washed again. The visualization was done via the chemiluminescence reaction using the Pro-light HRP Chemiluminescent Kit (PA112, Tiangen, Beijing, China) and Automatic Chemiluminescence Analysis (Tanon5200, Tianneng, Shanghai, China).

### 4.4. Cell Proliferation Analysis

The mouse spermatogonia cells before and after *Tle6*-KO were split into cell suspensions using Trypsin-EDTA (0.25%, Invitrogen, Carlsbad, CA, USA), and after counting, 5000 cells were inoculated into 96 well plates. Each group was repeated four times and placed at 37 °C, 5% CO_2_ saturated humidity, and cultivated in an incubator. After the cells had adhered to the wall, the cell viability was evaluated by measuring the 490 nm absorbance of the MTS reagent (G3582, Promega, Madison, WI, USA), 20 μL of which was added to each well, and the cells were incubated for 1 to 4 h in an incubator. Subsequently, five time points (24 h, 48 h, 72 h, 96 h, 120 h, 144 h, and 168 h) were selected to periodically detect the cell absorbance values, and draw a cell growth curve.

### 4.5. Cell Cycle and Cell Apoptosis Analysis

First, the mouse spermatogonia before and after *Tle6*-KO were made into a cell suspension with 0.25% Trypsin-EDTA, washed twice with prechilled DPBS, and then resuspended with 0.8 mL of prechilled DPBS. Subsequently, one tube of cells was taken as a negative control for each group of samples, and 5 μL of annexin-V (final concentration of 5 μg/mL, Beyotime, Shanghai, China), PI (final concentration of 15 μg/mL, Sigma), and annexin-V and PI, dyed at 4 °C for 20 min. Finally, 0.5 mL of cold DPBS was added to each tube of sample, and flow cytometry was used to adjust the compensation and to detect apoptosis.

Similarly, the mouse spermatogonia were digested, washed, and resuspended before and after *Tle6*-KO. Subsequently, one tube of cells was taken as a negative control for each group of samples, and 25 μL PI and 10 μL RNaseA (Thermo) were added to the other tube and placed in the dark at 37 °C for 30 min. Finally, 0.5 mL of cold DPBS was added to each tube sample, and the cell cycle was detected by flow cytometry.

### 4.6. Real-Time Quantity PCR Assay

After digesting the mouse spermatogonia before and after *Tle6*-KO, we extracted the total RNA using the microcell RNA extraction kit (74104, Qiagen), and used the PrimeScript ™ RT reagent Kit with gDNA Eraser (RR047Q, TaKaRa) to synthesize cDNA. Using the cDNA as a template, the Power SYBR Green RT-PCR Kit (Toyobo) was used to measure the relative expression levels of cell-proliferation- and cell-cycle-related genes (see Supplementary Table S1 for primer sequences) by qRT-PCR analysis on the Bio-Rad CFX96 PCR System (Bio-Rad). Each sample was repeated more than three times for each gene, and the test was repeated three times. Subsequently, using the GAPDH gene as a reference, we calculated the relative expression levels of genes related to cell proliferation and the cell cycle using the 2^−ΔΔCt^ (comparative cycle threshold) method. Finally, the Excel software was used for a *t*-test analysis of the quantitative results. When *p* > 0.05, the difference was not significant; when 0.01 ≤ *p* ≤ 0.05, the difference was significant; and when *p* < 0.01, the difference was extremely significant.

### 4.7. Statistical Analysis

All experiments were repeated at least three times, with similar results. GraphPad Prism version 5.0 (GraphPad Software, San Diego, CA, USA) was used for all statistical analyses. The differences between groups were assessed for statistical significance using Student’s *t*-test. The results are expressed as the means ± standard deviation (SD). *p* < 0.05 was considered to indicate a significant difference.

## 5. Conclusions

This study combined the CRISPR/Cas9 system and the Tet-on system to conditionally induce knockout of the mouse spermatogonia TLE6 protein to construct a mouse spermatogonia cell line with induced *Tle6* gene knockout. We found that the loss of the *Tle6* gene significantly affected the proliferation and cell cycle of spermatogonia, and, thus, provided a cellular model and scientific basis for studying the function of the *Tle6* gene on spermatogenesis.

## Supplementary Materials

Supplementary materials can be found at https://www.mdpi.com/1422-0067/21/16/5827/s1.

## References

[1] Wang W.F., Wang Y.G., Reginato A.M., Plotkina S., Gridley T., Olsen B.R. (2002). Growth defect in Grg5 null mice is associated with reduced Ihh signaling in growth plates. Dev. Dyn..

[2] Wang W., Wang Y.-G., Reginato A.M., Glotzer D.J., Fukai N., Plotkina S., Karsenty G., Olsen B.R. (2004). Groucho homologue Grg5 interacts with the transcription factor Runx2–Cbfa1 and modulates its activity during postnatal growth in mice. Dev. Boil..

[3] Zhang X., Chen H.-M., Jaramillo E., Wang L., D’Mello S.R. (2008). Histone deacetylase-related protein inhibits AES-mediated neuronal cell death by direct interaction. J. Neurosci. Res..

[4] Dang J., Inukai T., Kurosawa H., Goi K., Inaba T., Lenny N.T., Downing J.R., Stifani S., Look A.T. (2001). The E2A-HLF Oncoprotein ActivatesGroucho-Related Genes and SuppressesRunx1. Mol. Cell. Boil..

[5] Li D., Roberts R. (2001). WD-repeat proteins: Structure characteristics, biological function, and their involvement in human diseases. Cell. Mol. Life Sci..

[6] Marcal N., Patel H., Dong Z., Belanger-Jasmin S., Hoffman B., Helgason C.D., Dang J., Stifani S. (2005). Antagonistic effects of Grg6 and Groucho/TLE on the transcription repression activity of brain factor 1/FoxG1 and cortical neuron differentiation. Mol. Cell Biol..

[7] Bebbere D., Ariu F., Bogliolo L., Masala L., Murrone O., Fattorini M., Falchi L., Ledda S. (2014). Expression of maternally derived KHDC3, NLRP5, OOEP and TLE6 is associated with oocyte developmental competence in the ovine species. BMC Dev. Boil..

[8] Li L., Baibakov B., Dean J. (2008). A Subcortical Maternal Complex Essential for Preimplantation Mouse Embryogenesis. Dev. Cell.

[9] Yu X.-J., Yi Z., Gao Z., Qin D., Zhai Y., Chen X., Ou-Yang Y., Wang Z.-B., Zheng P., Zhu M.-S. (2014). The subcortical maternal complex controls symmetric division of mouse zygotes by regulating F-actin dynamics. Nat. Commun..

[10] Alazami A.M., Awad S.M., Coskun S., Al-Hassan S., Hijazi H., Abdulwahab F.M., Poizat C., Alkuraya F.S. (2015). TLE6 mutation causes the earliest known human embryonic lethality. Genome Boil..

[11] Otto T., Sicinski P. (2017). Cell cycle proteins as promising targets in cancer therapy. Nat. Rev. Cancer.

[12] Tchakarska G., Sola B. (2019). The double dealing of cyclin D1. Cell Cycle.

[13] Deltcheva E., Chylinski K., Sharma C.M., Gonzales K., Chao Y., Pirzada Z.A., Eckert M.R., Vogel J., Charpentier E. (2011). CRISPR RNA maturation by trans-encoded small RNA and host factor RNase III. Nature.

[14] Bai J., Li J., Mao Q. (2013). Construction of a Single Lentiviral Vector Containing Tetracycline-Inducible Alb-uPA for Transduction of uPA Expression in Murine Hepatocytes. PLoS ONE.

[15] Bai Y., Zhu C., Feng M., Pan B., Zhang S., Zhan X., Chen H., Wang B., Li J. (2020). Establishment of A Reversibly Inducible Porcine Granulosa Cell Line. Cells.

[16] Liu G., Liu K., Wei H., Li L., Zhang S. (2016). Generation of porcine fetal fibroblasts expressing the tetracycline-inducible Cas9 gene by somatic cell nuclear transfer. Mol. Med. Rep..

[17] Chen P.-C., Kuraguchi M., Velasquez J., Wang Y., Yang K., Edwards R., Gillen D., Edelmann W., Kucherlapati R., Lipkin S.M. (2008). Novel Roles for MLH3 Deficiency and TLE6-Like Amplification in DNA Mismatch Repair-Deficient Gastrointestinal Tumorigenesis and Progression. PLoS Genet..

[18] Verginelli F., Perin A., Dali R., Fung K.H., Lo R., Longatti P., Guiot M.-C., Del Maestro R.F., Rossi S., Di Porzio U. (2013). Transcription factors FOXG1 and Groucho/TLE promote glioblastoma growth. Nat. Commun..

[19] Welte K.H. (2014). G-CSF: Filgrastim, lenograstim and biosimilars. Expert Opin. Boil. Ther..

[20] Miyamoto M., Natsume H., Satoh I., Ohtake K., Yamaguchi M., Kobayashi D., Sugibayashi K., Morimoto Y. (2001). Effect of poly-l-arginine on the nasal absorption of FITC-dextran of different molecular weights and recombinant human granulocyte colony-stimulating factor (rhG-CSF) in rats. Int. J. Pharm..

[21] Kotzur T., Benavides-Garcia R., Mecklenburg J., Sanchez J.R., Reilly M.A., Hermann B.P. (2017). Granulocyte colony-stimulating factor (G-CSF) promotes spermatogenic regeneration from surviving spermatogonia after high-dose alkylating chemotherapy. Reprod. Boil. Endocrinol..

[22] Begay V., Baumeier C., Zimmermann K., Heuser A., Leutz A. (2018). The C/EBPbeta LIP isoform rescues loss of C/EBPbeta function in the mouse. Sci. Rep..

[23] Schuster M.B., Porse B.T. (2006). C/EBPalpha: A tumour suppressor in multiple tissues?. Biochim. Biophys. Acta..

[24] Chen S.S., Chen J.F., Johnson P.F., Muppala V., Lee Y.H. (2000). C/EBPbeta, when expressed from the C/EBPalpha gene locus, can functionally replace C/EBPalpha in liver but not in adipose tissue. Mol. Cell Biol..

[25] Jones L.C., Lin M.-L., Chen S.-S., Krug U., Hofmann W., Lee S., Lee Y.-H., Koeffler H.P. (2002). Expression of C/EBPbeta from the C/ebpalpha gene locus is sufficient for normal hematopoiesis in vivo. Blood.

[26] Hoffman B.G., Zavaglia B., Beach M., Helgason C.D. (2008). Expression of Groucho/TLE proteins during pancreas development. BMC Dev. Boil..

[27] Duncan F.E., Padilla-Banks E., Bernhardt M.L., Ord T.S., Jefferson W.N., Moss S.B., Williams C.J. (2014). Transducin-Like Enhancer of Split-6 (TLE6) Is a Substrate of Protein Kinase A Activity During Mouse Oocyte Maturation1. Boil. Reprod..

[28] Zhu K., Yan L.-Y., Zhang X., Lu X., Wang T., Liu X., Qiao J., Li L. (2014). Identification of a human subcortical maternal complex. Mol. Hum. Reprod..

[29] Swanton C. (2004). Cell-cycle targeted therapies. Lancet Oncol..

[30] Bochis O.V., Irimie A., Pichler M., Neagoe I.B. (2015). The Role of Skp2 and its Substrate CDKN1B (p27) in Colorectal Cancer. J. Gastrointest. Liver Dis..

[31] Malumbres M., Barbacid M. (2001). To cycle or not to cycle: A critical decision in cancer. Nat. Rev. Cancer.

[32] Zhang W., Cao L., Sun Z., Xu J., Tang L., Chen W., Luo J., Yang F., Wang Y., Guan X. (2016). Skp2 is over-expressed in breast cancer and promotes breast cancer cell proliferation. Cell Cycle.

[33] Kowalska E., Bartnicki F., Fujisawa R., Bonarek P., Hermanowicz P., Tsurimoto T., Muszyńska K., Strzalka W. (2018). Inhibition of DNA replication by an anti-PCNA aptamer/PCNA complex. Nucleic Acids Res..

[34] Wang T.C., Wei J.J., Sabatini D.M., Lander E.S. (2013). Genetic Screens in Human Cells Using the CRISPR-Cas9 System. Science.

[35] Cheng T., Xue X., Fu J. (2014). Effect of OLIG1 on the development of oligodendrocytes and myelination in a neonatal rat PVL model induced by hypoxia-ischemia. Mol. Med. Rep..

