# Supplementary material for: Knockout of the Transducin-Like Enhancer of Split 6 Gene Affects the Proliferation and Cell Cycle Process of Mouse Spermatogonia
Source: Int J Mol Sci. 2020 Aug 13;21(16):5827. doi: 10.3390/ijms21165827 (PMC7461562; doi:10.3390/ijms21165827)
Supplement: Supplementary file 1 [file ijms-21-05827-s001.pdf]

**Supplementary Table 1.** The sequence of primer.

| Gene | Use | | Primer Sequence (5′ - 3′) | Product Size (bp) |
|---|---|---|---|---|
| mU6 | Vector | F | TTTGGCGCCGGCTCGAGTGTACA | 375 bp |
| | | R | AAACAAGGCTTTTCTCCAAGGG | |
| *Tle6*-SgRNA | Vector | F | TGGAGAAAAGCCTTGTTTgGAGGCCTTCGACCAA GTGCTGTTTTAGAGCTAGAAATAGC | 163 bp |
| | | R | CACCGGTTAGCGCTAGCTAATGCC | |
| *Tle6* | Genome | F | CAGAACAATCGGCTACATAGACCC | 728 bp |
| | | R | GCCAGCAATCGTTTGAAGACA | |
| M13 | Genome | F | GTAAAACGACGGCCAG | 728 bp |
| | | R | CAGGAAACAGCTATGAC | |
| *Gadph* | qRT-PCR | F | TGGTGAAGGTCGGTGTGAAC | 231 bp |
| | | R | GCTCCTGGAAGATGGTGATGG | |
| *Cdk 2* | qRT-PCR | F | CGGATCTTTCGGACTCTGGG | 250 bp |
| | | R | GAAGGTGGGGCACTGGTTTA | |
| *Cdk 4* | qRT-PCR | F | GGAAACTCTGAAGCCGACCA | 245 bp |
| | | R | TTGTGCAGGTAGGAGTGCTG | |
| *Cyclin D1* | qRT-PCR | F | TTTGGCAGAAGGTGGAGAGC | 190 bp |
| | | R | GACCCTCCAATAGCAGCGAA | |
| *Cyclin E* | qRT-PCR | F | GCTTCGGGTCTGAGTTCCAA | 208 bp |
| | | R | GCTGACTGCTATCCTCGCTT | |
| *Pcna* | qRT-PCR | F | TGGTAGTTGTCGCTGTAGGC | 161 bp |
| | | R | CCTTCTTCAGGATGGAGCCC | |
| *Skp2* | qRT-PCR | F | AGGCAGGACTACGAGCTAGG | 188 bp |
| | | R | TGTTTTAGCGCTCGGCTTAGA | |
| *C/ebp α* | qRT-PCR | F | AGCTACAGGGAGGTGGAGG | 244 bp |
| | | R | ACACAAGGCTAATGGTCCCC | |
| *C/ebp β* | qRT-PCR | F | GGGCCCTGAGTAATCACCTTAAA | 204 bp |
| | | R | TCTTCACTTTAATGCTCGAAACGG | |
| *G-csf* | qRT-PCR | F | CCCTGGAGCAAGTGAGGAAG | 227 bp |
| | | R | TAGAGCCTGCAGGAGACCTT | |

F, forward primer; R, reverse primer.

**Supplementary Table 2.** Primary and secondary antibodies used for western blot analysis.

| Antibody | Dilution | Supplier |
|---|---|---|
| Anti-TLE6 (D-4): mouse IgG | 1: 500 | Santa Cruz, Heidelberg (sc-515065) |
| Anti-GAPDH: mouse IgG | 1:1000 | Transgen, China (HC301) |
| Anti-FLAG: mouse IgG2b | 1:1000 | Absin, China (abs830005a) |
| Goat Anti-Mouse IgG(H+L), HRP Conjugate | 1:1000 | Transgen, China (HS201) |

**Supplementary Table 3.** The statistics of *Tle6* knockout efficiency.

| Total Sequencing | Insertion Mutation | Number of Mutations Missing | Number of Wild Type Sequences | Knockout Efficiency（%） |
|---|---|---|---|---|
| 24 | 11 | 10 | 3 | 87.5 |
